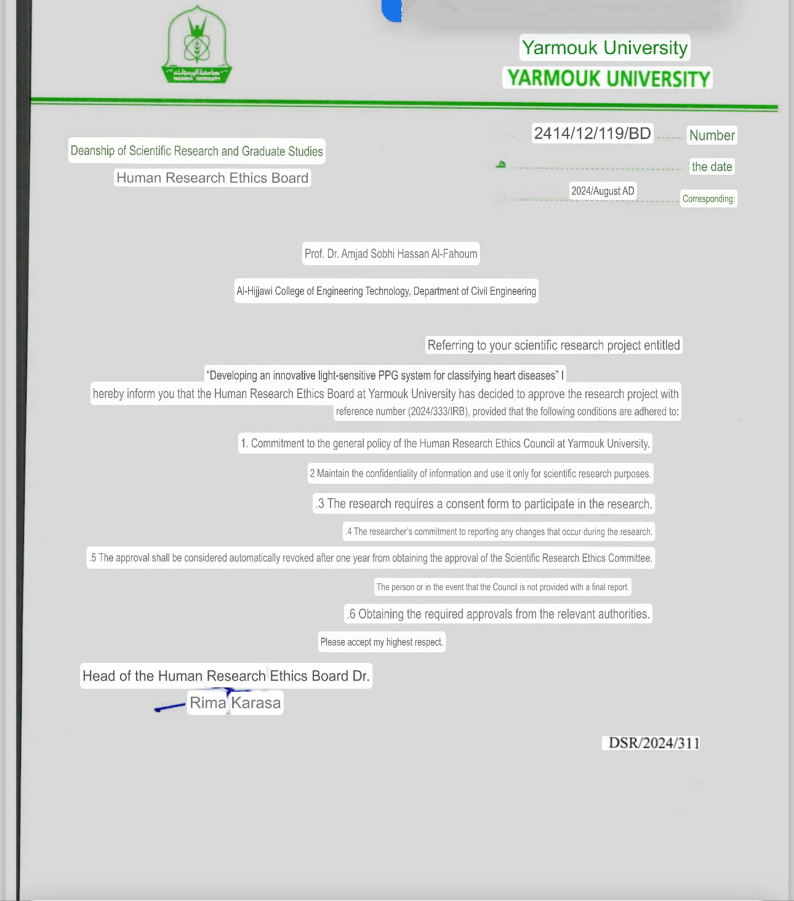# Development of a novel light-sensitive PPG model using PPG scalograms and PPG-NET learning for non-invasive hypertension monitoring

**DOI:** 10.1016/j.heliyon.2024.e39745

**Published:** 2024-10-23

**Authors:** Amjed Al Fahoum, Ahmad Al Omari, Ghadeer Al Omari, Ala'a Zyout

**Affiliations:** Biomedical systems and Informatics Engineering Dept., Hijjawi Faculty for Engineering Technology, Yarmouk University, Irbid, 21163, Jordan

**Keywords:** Continuous wavelet transform (CWT), Photoplethysmography (PPG), Deep learning, Hypertension classification, And PPG-NET architecture

## Abstract

**Background and objective:**

Photoplethysmography (PPG) signals provide a non-invasive method for monitoring cardiovascular health, including blood pressure levels, which are critical for the early detection and management of hypertension. This study leverages wavelet transformation and special purpose deep learning model, enhanced by signal processing and normalization, to classify blood pressure stages from PPG signals. The primary objective is to advance non-invasive hypertension monitoring, improving the accuracy and efficiency of these assessments.

**Methods:**

The study employed continuous wavelet transform (CWT) to prepare PPG signals for analysis using a special purpose PPG-NET designed by applying advanced deep-learning models. PPG-NET was verified by applying several pre-trained models, including Inception, MobileNetV2, InceptionResNetV2, and others to the PPG data. Rigorously five-fold cross-validated models were conducted to obtain the models' performance to ensure robustness and repeatability of results.

**Results:**

The PPG-NET model demonstrated superior performance, achieving a perfect accuracy of 100 % in classifying the four stages of hypertension—normal, prehypertension, stage 1, and stage 2. The evaluation metrics reported include precision, sensitivity, and specificity, with the PPG-NET model achieving 100 % across all metrics. Other models showed varying levels of accuracy, with InceptionV3 also reaching 91.5 %, while some, like VGG-19, underperformed significantly.

**Conclusions:**

Integrating CWT and PPG-NET offers a promising avenue for enhancing non-invasive blood pressure monitoring. The PPG-NET model, in particular, showed potential for clinical application due to its high accuracy and reliability. This study showed the effectiveness of combining advanced computational techniques with traditional PPG analysis, potentially leading to more personalized and accessible hypertension management strategies.

## 1 Introduction

Cardiovascular diseases (CVD) affect the heart and blood vessels and pose worldwide health risks [1]. CVDs top the global mortality toll at 17.9 million. CVDs include rheumatic heart disease, coronary heart disease, and cerebrovascular disease. Heart attacks and strokes cause more than four out of five CVD fatalities, and one-third of them occur prematurely in people under 70 [2]. Hypertension occurs when blood vessel pressure (BP) is 140/90 mmHg or above. Hypertension is a leading cause of premature death worldwide. Two-thirds of the 1.28 billion adults aged 30–79 with hypertension live in low- and middle-income countries. Only 42 % of adult hypertensive symptoms are diagnosed and treated [3]. Photoplethysmography (PPG) measures tissue microvascular blood volume [4]. PPG works largely non-invasively at red or near-infrared wavelengths. Basic PPG technology requires only a light source to illuminate skin tissue and a photodetector to detect minute changes in light intensity due to catchment volume perfusion [5]. Too much blood pressure can harm the heart, kidneys, and other organs, creating several issues. Therefore, early hypertension detection, management, and control can considerably improve CVD prevention and treatment. Healthcare has entered a new era. The strong computer technology of deep learning has addressed several identification and classification problems [6]. Deep learning (DL) surpasses standard machine learning by extracting high-level characteristics from enormous data sets. It extracts data features automatically using multilayer feature extraction and unsupervised feature learning.

Compared to normal machine learning, deep learning requires a lot more training data. It takes a lot of data to find deep patterns. In certain specialties, the absence of sufficient training data poses a significant challenge. Data collection is complicated and expensive, making it difficult to create a complete, high-quality annotated dataset. Transfer learning in deep learning can modify the condition of the same distribution and independence between training and test data to overcome insufficient training data. Deep transfer learning extracts knowledge from many fields using deep neural networks [7]. Photoplethysmography (PPG) signals from non-invasive optical technologies are increasingly useful for cardiovascular health monitoring [8]. Advanced deep learning (DL) algorithms have improved PPG-based systems' classification and estimation of diseases, including high blood pressure, a frequent heart issue [9]. Studies have investigated ways to determine blood pressure from PPG signals using technology. Deep learning models, signal processing, and hybrid approaches using PPG data and its first and second derivatives are used [10]. In some studies, LSTM models predict the constant blood pressure in the arteries, while convolutional neural networks identify the observed PPG signals [10,11]. In other studies, blood pressure measurement accuracy was correlated with PPG signal variations. Gao et al. tested a large model sample, demonstrating promising accuracy and real-world application [12]. Panwar et al.'s PP-Net accurately predicts heart rate and blood pressure in physiological conditions [13]. Ali and Atef estimated blood pressure from PPG data using a multi-stage LSTM network. They mainly classified blood pressure as norm tension and hypertension. The model's blood pressure estimation accuracy suggests clinical application [14]. Using PPG and RPPG signals, Shrumpf et al. predicted blood pressure with RNNs. They used comprehensive feature vectors to improve accuracy across blood pressure ranges, showing how transfer learning can optimize PPG signal processing [15]. Recently, Chu et al. created a British Hypertension Society-compliant deep learning system with excellent accuracy. High criteria are crucial for building medical device software for non-invasive monitoring [16]. González et al. published benchmark studies on non-invasive blood pressure prediction from PPG signals, emphasizing the need to develop these technologies despite encouraging first results [17]. Wang et al. developed a data-driven, deep-learning-based end-to-end solution that leverages transfer learning to estimate blood pressure from short PPG signals using a CNN trained on ImageNet for picture categorization. Images were created from PPG segments using a visibility graph (VG). VG image feature vectors were extracted using a pre-trained deep CNN. Ridge regression solved the feature vector-reference BP weights and biases [18]. By matching the type of continuous wavelet transform (CWT) and segment length, Wu et al. came up with a very accurate way to predict BP categorization from PPG using CNN and CNN-GoogleNet. The Cgau1 wavelet and segment-300 could classify normal and abnormal blood pressure with 90 % accuracy [19]. Al Fahoum et al. utilized CWT to extract PPG characteristics, then transferred this learning to pre-trained deep learning models, such as InceptionV3, VGG-16, and ResNet101, to classify blood pressure readings into hypertension stages 1 and 2, normal, and pre-hypertension. InceptionV3, VGG-16, and ResNet101 had 99.5 %, 22.5 %, and 92.5 % accuracy [9]. Pankaj et al. updated the last three layers of GoogleNet, DenseNet, and AlexNet to divide the PPG signal's temporal frequency (TF) spectrogram into three groups: norm tension, prehypertension, and hypertension. For five-fold training and testing, GoogleNet had 94.81 % accuracy, DenseNet 96.51 %, and AlexNet 95.21 %. For PPG feature extraction, multiple types of CWT were used to build images, which are the core of classification [19]. CWT can also detect BP risk [20]. PPG-only deep learning algorithms were proposed by Cano et al. to assess BP dangers. CWT PPG signal scalograms were sent to GoogleNet and ResNet for categorization [21]. Liang et al. tested whether deep learning might improve hypertension risk categorization using PPG signals based on the continuous wavelet transform (Morse) [22]. Due to physical activity, skin type, and environmental factors, PPG signals, deep learning, and transfer learning are challenging to monitor high blood pressure. Improved monitoring should be reliable and continuous. These methods using wearable devices could improve hypertension management and personalized healthcare. In this paper, processing, scalograms, and deep learning-based PPG-NET improve worldwide cardiovascular health monitoring and management. The use of continuous wavelet transform (CWT) with the PPG-NET architecture enhances the classification of blood pressure from PPG data. Deep learning was used to categorize PPG-based BP. However, CWT and PPG-NET made it easier for the model to obtain time-frequency domain information needed for PPG signals. This improves performance and measurement accuracy. Real-time wearable technology applications benefit from PPG-NET's depth wise separable convolutions' precision and low processing cost. Scalograms extracted more complex information, making BP fluctuations easier to present and identify than raw PPG signals or more straightforward alterations. The proposed new architecture correctly identifies all stages of high blood pressure, even with fewer patients like Stage 2, which was wrongly labeled in earlier studies because of too much sampling from an imbalanced dataset. This work promotes noninvasive measurements and tailored medicine, predicting future reliable healthcare solutions. The reminder of the paper is structured as follows: Methodology includes experimental setup, data gathering, model theory, and training. Results show the performance of the deep learning approach and its comparisons. These data are interpreted using authentic literature and future biometric variable integration. The paper summarizes its findings and calls for more research on hypertension monitoring and healthcare technology.

## 2 Materials and methods

**Fig. 1:**
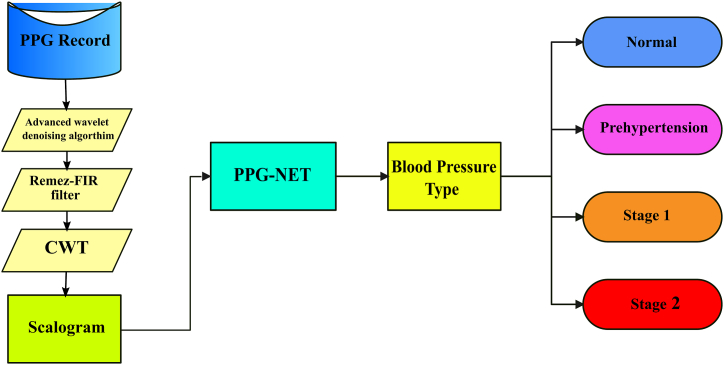
Overall basic flowchart of the proposed study.

This research approach can be summarized as follows: first, PPG records of various kinds were gathered; next, the PPG data was prepared using a preprocessing stage; third, CWT was created for the data to transform the PPG signal into various images for hypertension classes; and finally, the images were fed into a uniquely designed deep learning network called PPG-NET to obtain the optimal model specifications and accomplish classification among four classes. The implemented approach for PPG signal classification is divided into three main steps, as shown in Fig. 1.

### 2.1 Dataset and hypertension levels

The signals were used to show the level of BP and confirm its classification. The records were divided into 4 classes: normal, prehypertension, stage 1 hypertension, and stage 2 hypertension. The data used to validate this study is obtained from Guilin People Hospital and Guilin University of Electronic Technology in China [23]. The dataset comprises 657 PPG segments from 219 subjects aged 21 to 86 (median age 58), with 48 % male. The dataset includes individuals with hypertension, diabetes, cerebral infarction, and insufficient brain blood supply, representing various BP categories: normal, prehypertension, stage 1 hypertension, and stage 2 hypertension. A custom portable hardware platform with a SEP9AF-2 PPG sensor probe, an MSP430FG4618 microcontroller, and a Bluetooth-enabled app was used to record PPG signals continuously for three 2.1-s periods per subject at a sampling frequency of 1000 Hz [23]. The hardware had a 0.5–12 Hz bandpass filter and utilized dual LEDs at 660 nm (red) and 905 nm (infrared) wavelengths [23]. The recordings were segmented into 2.1-s intervals for easier CWT processing and to enhance the deep learning model’s ability to classify blood pressure stages. In addition to PPG data, cuff-based BP measurements were taken at intervals for ground truth. Data collection involved capturing basic physiological information, cardiovascular disease data from medical records, and simultaneous PPG and BP readings. Subjects sat relaxed, collecting PPG from the left index finger and BP from the right forearm, all within 3 min. Each PPG segment was saved only if its Skewness SQI value exceeded zero, minimizing noise and artifacts. Nurses classified patients into BP categories using cuff-based measurements, and clinical experts verified their work. Non-normal BP subjects were confirmed to be in a continuous non-normal BP state during recording, ensuring the accuracy and reliability of the data for training and testing.

Per Yarmouk University's ethical committee's directives, a distinct dataset was collected at King Abdullah University Hospital under medical supervision. The study's Institutional Review Board number is (IRB/2024/333).

### 2.2 Dataset preprocessing

Accurately detecting events and evaluating physiological indicators requires acquiring high-quality, low-noise PPG signals, which enhance health monitoring, screening, and diagnosis techniques [22]. The advanced adaptive wavelet denoising algorithm is a complex signal processing method that improves the quality of PPG signals [24]. Motion artifacts, ambient light interference, and electronic equipment noise often affect PPG signals, which monitor changes in blood volume in the microvascular tissue bed [25]. To address these issues, the adaptive wavelet denoising algorithm utilizes the wavelet transform to analyze various PPG frequencies with a resolution that matches each scale [24]. The algorithm works by decomposing the PPG signal into different levels of detail using wavelets. At each level, it adaptively thresholds the wavelet coefficients, effectively isolating and removing the noise while retaining the pivotal features of the original signal. This method is particularly adept at handling non-stationary noise components, which are common in PPG signals due to their physiological and environmental variability.

The Finite Impulse Response (FIR) Remez filter further processes the PPG signal after the denoising step. We use the Remez filter design algorithm, also referred to as the Parks-McClellan algorithm, to generate an optimal bandpass filter that satisfies particular requirements [26]. The FIR filter tailors itself to allow a frequency band from 0.05 to 16 Hz to pass through for PPG signals. This frequency range is chosen to include the heart rate and respiratory rate information while excluding higher-frequency noise that is not relevant to the physiological measurements.

**Fig. 2:**
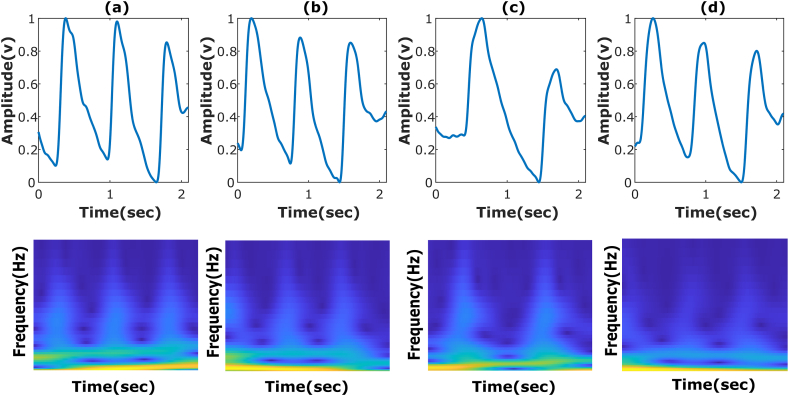
The first row are samples of the preprocessed PPG types, and the second are the CWT representations: (a) Normal case; (b) Prehypertension case; (c) Stage 1 of hypertension case; (d) Stage 2 of hypertension case. The x-axis for both rows represents time (in seconds), and the y-axis represents the amplitude of the PPG signal (top row) and the frequency (in Hz) for the CWT scalogram (bottom row).

Finally, the signal is normalized using a 0–1 min-max normalization technique. This process rescales the amplitude of the PPG signal to a standard range of values between 0 and 1. Normalization reduces the differences among signals and makes the process of feature extraction and analysis more reliable. By doing so, the algorithm ensures that the PPG signal is not only free from noise but also standardized for consistent analysis across various conditions and applications [27]. The output filter was applied to the PPG signal shown in Fig. 2, the first row.

### 2.3 PPG signal transformation

A signal can be transformed into a different form using the wavelet transform. The purpose of this transformation is to depict the original signal and expose the characteristics or features that were previously buried within it. The wavelet transform cannot be performed without a basis wavelet [[28], [29], [30]]. The definition of the signal x (t) with the scaled or shifted forms from a base wavelet $\varphi_{a,b}\left(t\right)$ is the definition of the CWT for the signal x (t).

(1) $$CWT\left(a,b\right)=\frac{1}{\surd a}\int_{-\infty }^{+\infty }x\left(t\right)*\varphi_{a,b}\left(\frac{t-b}{a}\right)dt$$

**a:** dilatation**, a** ∈ $R^{+}-$ {0}

**b**: translation parameter**, b ∈ R,**

The CWT is the product of scaled and shifted forms from a mother wavelet φ(t) and the sum of input signal x(t) [31].

(2) $$CWT\left(scale,position\right)=\int_{-\infty }^{+\infty }x\left(t\right)*\varphi \left(scale,position,t\right)dt$$

Frequency-Time analysis enables us to understand how the signal is distributed with frequency and time [32]. The Morlet wavelet is used for conducting the CWT to locate transitory elements present in signals, using the optimum time-frequency bandwidth [30]. The Morlet wavelet ψ, also known as the Gabor wavelet, is denoted by Ref. [32]:

(3) ψMorlet(t)=e−ωo2(t22)ejωot

For this work, the analytic Morlet (Gabor) wavelet was utilized to perform the CWT. MATLAB® generates the CWT coefficients using a filter bank with a sampling frequency of 1000 Hz. For each type of PPG signal, a salogram image representation was generated. Fig. 2 illustrates this representation, with the processed signal displayed in the first row and the final images representing each class in the second row. Subsequently, the saved scalograms were fed next into the designed deep learning models, as exemplified in Fig. 1.

### 2.4 Data oversampling

Classification problems in extended supervised learning involve imbalanced datasets, where algorithms often bias towards the majority classes, leading to higher misclassification rates for the minority classes. Researchers have proposed various approaches to address this issue, including cost-sensitive learning, algorithmic changes, and data sampling. Oversampling techniques, were employed to balance the dataset by increasing the number of instances in underrepresented classes. In contrast, undersampling was used to reduce the number of instances in overrepresented classes, ensuring that the model is trained on a balanced dataset without bias toward any specific class [33]. The oversampling method was used in this paper to address the issue of imbalanced data. Oversampling is used to increase the number of data points in underrepresented Stage 1 and Stage 2 hypertension patients, ensuring that the dataset is balanced. According to current data, the Synthetic Minority Oversampling Technique (SMOTE) creates synthetic samples for Stage 1 and Stage 2 hypertension. To create synthetic data points, SMOTE selects minority data points and interpolates them with their nearest neighbors. This approach generates credible data instances that are not duplicates but similar to the original samples. Using SMOTE, Stage 1 and Stage 2 data were included without overfitting the model to duplicate data points. In this work, SMOTE was essential for giving the model enough data to learn from all blood pressure phases. The dataset was balanced by adding Stage 1 and Stage 2 examples, allowing the algorithm to reliably categorize hypertension stages without favoring normal and prehypertension. This method assures that the classifier can generalize effectively across all categories, especially the hardest stages, improving minority class prediction performance and accuracy. To validate the proposed approach, the minimum of 60 is also used among the 4 classes to check if the model still performs correctly. Using synthetic data points from a subject for training and testing could leak data; thus, subject IDs were kept intact. Subject-aware splitting ensures that the model is tested on actually unseen data rather than overfitting to repeated subject data, preserving evaluation integrity. This method simulates better real-world settings by requiring the model to generalize across individuals rather than relying on subject-specific properties in training and testing. Table 1 shows the number of data segments in each class before applying oversampling techniques.

**Table 1:** Number of data for each class on the first row before applying data over sampling and second row after applying data over sampling.

Class name:	Normal	Prehypertension	Stage 1	Stage 2
	240	255	102	60
	250	250	250	250

The dataset used to train PPG-NET contains several samples from the same patients because the scalograms are created from short PPG segments. To create classification scalograms, each subject's PPG signal was separated into three segments. Second, the oversampling employed to balance the dataset increases the possibility of numerous data points from the same subject. Subject-aware data splits prevented overfitting and prevented the same subject from appearing in both training and test sets. This prevents similar patient data from appearing in the training and test sets. The authors made sure that all data from an individual, including original and oversampled data, was assigned to the training set or the test set, never both. The dataset was shuffled prior to training, but only in the training set after the subject-aware split. This method assures that the model's performance represents its capacity to generalize to new subjects rather than learning from redundant or overlapping data, which could lead to overoptimistic trial results.

### 2.5 PPG-NET: A deep dive into convolutional neural networks (CNNs)

CNNs have advanced the science of computer vision by demonstrating exceptional ability in tasks such as semantic segmentation, object identification, and picture categorization. However, the need for increasingly powerful and effective models has grown in parallel with the increasing complexity and number of datasets. As a result, the authors developed a customized CNN using advanced CNN structures, known as the PPG-Net.

#### 2.5.1 Development and motivation

**Fig. 3:**
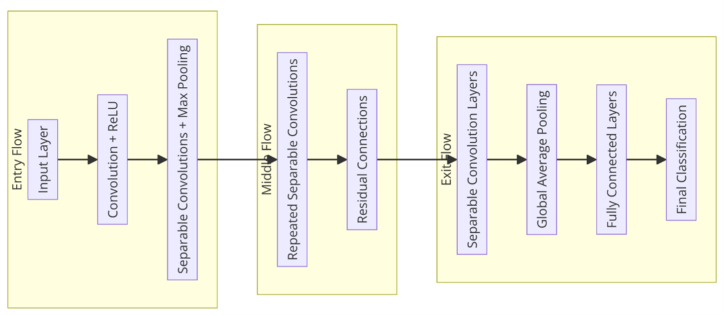
The main stages of the PPG-NET.

The PPG-Net, which can also refer to as a "light-based PPG characterization deep learning network," was developed to analyze scalograms generated for PPG signals. The intent of this architecture was to enhance the Xception and Inception designs, which had previously established new benchmarks for deep learning in terms of efficiency and accuracy. The primary goal of PPG-NET was to address the limitations of conventional CNNs and the Inception model by creating a more efficient and robust architecture that will reduce computational expenses. The primary improvement in PPG-NET is the implementation of depth wise separable convolutions, a technique that was initially introduced by the Inception design and is now exploited to its fullest potential in PPG-NET. Traditional convolutions are computationally expensive because they simultaneously convolve the spatial and depth dimensions of an input tensor. Conversely, depth wise separable convolutions partition this process into two distinct stages: pointwise convolution and depth wise convolution. Depth wise convolution is a method in which a single convolutional filter is applied to each input channel. This helps to preserve spatial information and reduces the amount of processing required. Pointwise convolution is the process of combining the results of depth wise convolution across multiple channels using a 1x1 convolution. By segregating these processes, PPG-NET reduces parameters and computational costs significantly while simultaneously maintaining or improving the network’s performance. Fig. 3 shows the main stages of the PPG-NET.

#### 2.5.2 Architecture and structure

**Fig. 4:**
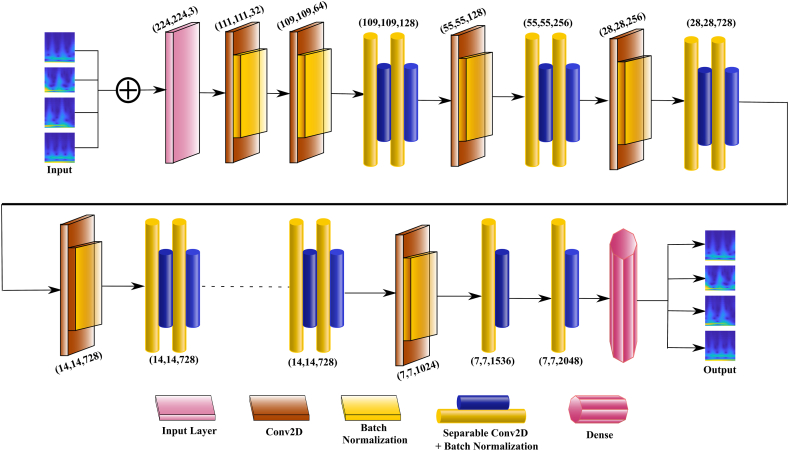
The architecture of the PPG-NET, the input is the scalogram image and the output is the classification of the level of BP.

The PPG-NET architecture shown in Fig. 3 distinguishes itself from the ResNet and Inception models by replacing conventional Inception modules with depth wise separable convolutions, a notable advancement. Its composition can be divided into three main components. There are several convolutional layers in the entry flow. These are followed by depth wise separable convolutions and max pooling layers, which make the input image smaller. Middle Flow is made up of multiple residual blocks, each of which contains depth-wise separable convolutions. The blocks are linked together via shortcut connections, which facilitates network training with increased depth. During the exit flow, the feature maps are subjected to further processing by depth wise separable convolutions. The technique is finalized by incorporating a global average pooling layer and a fully linked layer, both of which serve the classification purpose. Fig. 4 shows the detailed architecture of the proposed PPG-NET.

PPG-NET is computationally efficient because it uses depth-wise separable convolutions. By decoupling the spatial and depth convolutions, the model reduces the number of parameters and the amount of computation required. For instance, a standard convolutional layer with Dk × Dk × M × N parameters can be replaced by a depth wise separable convolution with parameters, where Dk is the kernel size, M is the number of input channels, and N is the number of output channels. This significantly reduces the number of parameters and the computational load. PPG-NET is characterized by its efficiency and scalability, making it suitable for this range of applications, from image classification and object detection to more complex tasks like image segmentation and generative modeling. Its reduced computational cost and high performance make it an optimum choice for deployment in resource-constrained environments, such as home-based microcontroller applications, mobile devices, and embedded systems. The initial weights of the network were obtained first from the ImageNet [34]. To prevent data leakage, the training procedure followed a subject-aware 80/20 train/test split, ensuring there was no overlap between subjects in the training and test sets. A five-fold cross-validation was implemented on the training set to enhance generalization and mitigate overfitting, with each fold using four subsets for training and one for validation. Early stopping was implemented to avoid overfitting, halting training if validation performance plateaued. The model was trained using the cross-entropy loss function and optimized using the stochastic gradient descent (SGD) optimizer. The learning rate was set to 0.01, and the batch size was set to 12. There were 100 epochs. After training, the final model was evaluated on the 20 % test set, ensuring robust generalization and accurate performance assessment on new subjects. Once PPG-NET training demonstrated 100 % accuracy without any signs of overfitting, the final weights were saved and used later as initial weights for the PPG-NET.

#### 2.5.3 Deep learning models

To study the effectiveness of the PPG-NET as an optimized network for PPG data, several pre-trained deep learning models were used for classifying the four stages of hypertension. This study used the following pre-trained networks: MobileNetv2, DenseNet201, InceptionResNetV2, InceptionV3, VGG-19, ResNet50, and NASNetLarge to validate the performance of the proposed PPG-NET. These methods were applied while considering the size and variety of the dataset. The aim is to use various model types and pre-trained models to determine their effectiveness in classifying PPG related scalograms. As a result, they are limited to a particular group due to the numerous options available.

### 2.6 The method of evaluation

**Fig. 5:**
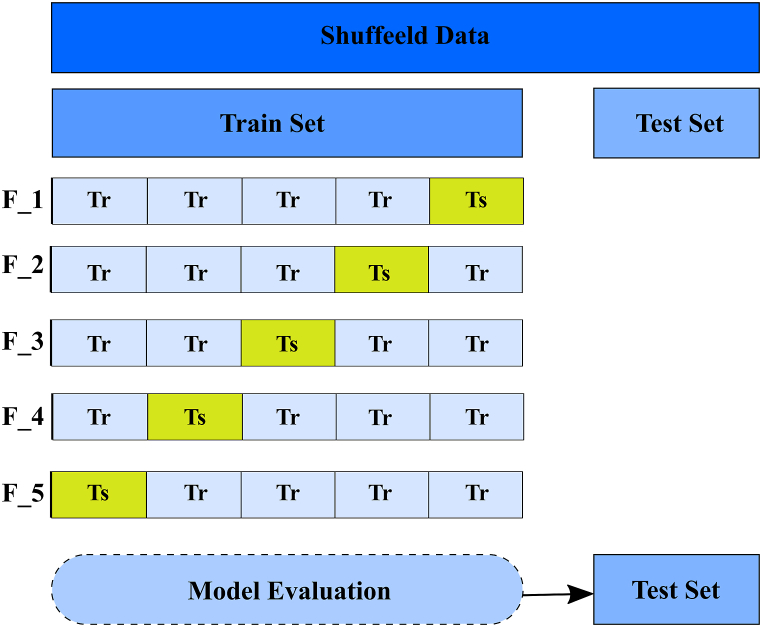
illustrates for model training/testing process, F: for Fold number, Tr for training set and Tr for resting set inside the fold.

A five-fold cross-validation approach is used to validate the experimental findings. In five-fold cross-validation, the dataset is divided into five equal-sized parts. One part was designated as the test set, and the remaining parts were used as the training set. The model was tested using the test set to estimate its metrics. The steps of five-fold cross-validation are illustrated in Fig. 5.

### 2.7 Metrics for evaluation

According to medical terminology, true positives (TP) indicate that patients with abnormal lesions are tested using a predetermined screening procedure and are appropriately classified as having abnormal lesions. A positive result, on the other hand, indicates that the patient had abnormal lesions or had the virus. Medical misdiagnosis of a patient without abnormal lesions is known as a false-positive (FP) diagnosis [26,35]. The likelihood that patients with anomalous lesions or viruses would be diagnosed as medically positive is called sensitivity (Sen.) or recall, commonly referred to as the true positive rate. This number should be as high as possible. The more sensitive the diagnosis, the greater the value. The likelihood that a patient will be identified as negative even in the absence of aberrant lesions is called specificity (Spe.) or true-negative rate. Similarly, this value should be as high as possible. In other words, the more accurate the diagnosis, the higher the value. The percentage of genuine samples to total samples is known as accuracy (Acc.). Sen. and Spe are the most precise and scientific standards for medical image evaluation. In this paradigm, higher sensitivity corresponds to lower rates of missed diagnoses, while higher specificity corresponds to lower rates of misdiagnosis [36]. The difference between the total number of samples predicted to be positive and the number of samples that are actually positive is known as precision (Pre.). The F1-score or sensitivity score is the harmonic mean of recall and precision [37]. The following equations represent the key performance metrics, rather than relying solely on textual descriptions:

(4) $$\text{Accuracy}=\frac{\text{TP}+\text{TN}}{\text{TP}+\text{TN}+\text{FP}+\text{FN}}$$

Where:

TP = True Positives.

TN = True Negatives.

FP= False Positives.

FN = False Negatives

(5) $$\text{Precision}=\frac{\text{TP}}{\text{TP}+\text{FP}}$$

Precision measures the proportion of true positive predictions among all positive predictions.

(6) $$\text{Recall}=\frac{\text{TP}}{\text{TP}+\text{FN}}$$

Recall calculates the proportion of actual positives that are correctly identified.

(7) $$F1=\frac{2\times \text{Precision}\times \text{Recall}}{\text{Precision}+\text{Recall}}$$

The F1-score is the harmonic mean of precision and recall, balancing the two metrics.

## 3 Results

**Fig. 6:**
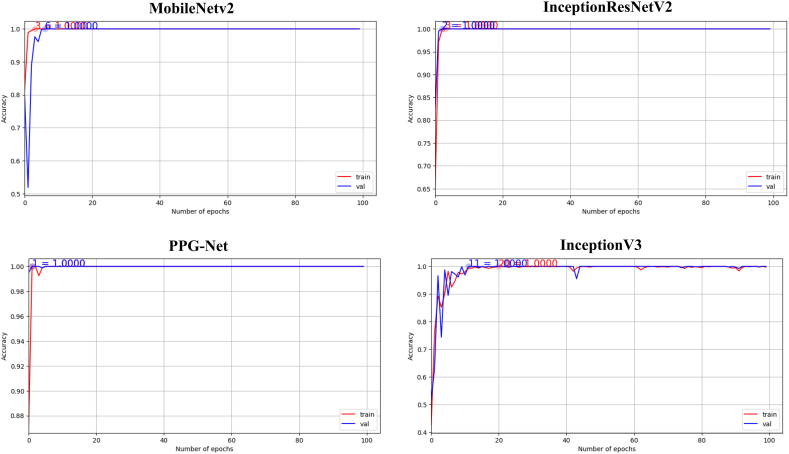
The progress of training and validation for four DL models: MobileNetv2, InceptionResNetV2, Xception, and InceptionV3.

This section presents the experimental findings of the proposed model. Fig. 4 shows the progress of training and validation for the four DL models: MobileNetv2, InceptionResNetV2, PPG-NET, and InceptionV3. It shows the training and validation scores of a model over several epochs. Each graph symbolizes a distinct metric or condition that evaluates the model. The red line represents the training score, and the blue line represents the validation score. The PPG-NET graph shows a model that achieves high accuracy quickly and maintains stability throughout the training process, which is indicative of good generalization and possibly an indication that early stopping could be effective. The uniformity of high performance across all metrics, as well as the similarity between the training and validation lines, suggests a well-performing model that generalizes well to unseen data. However, the InceptionV3 graph (Fig. 6) shows some fluctuations in the validation score, which could imply over-fitting or a need for further tuning.

Overall accuracy is shown in Table 2. For each class, other performance measures were calculated and shown respectively as Acc. Precision as Pre. in Table 3, sensitivity as Sen. in Table 4, and F1-score in Table 5. The different networks are used to classify non-invasive blood pressure (BP) using PPG signals to classify them in any of the four classes: normal (N) prehypertension (P), stage 1 hypertension (S1), and stage 2 hypertension (S2) based on pre-trained Xception, MobileNetv2, DenseNet201, InceptionResNetV2, InceptionV3, VGG-19, ResNet50, NASNetLarge, and the proposed PPG-NET.

**Table 2:** The overall Accuracy and the Accuracy (Acc.) of each class for all pre-trained models.

Classifier	Acc.				Over all Acc.
	N	P	S1	S2	
**PPG-NET**	100 %	100 %	100 %	100 %	**100 %**
**Xception**	84 %	82.00 %	96 %	98.00 %	**90 %**
**MobileNetv2**	84.00 %	78.00 %	92 %	96 %	**87.5 %**
**DenseNet201**	74.00 %	72.00 %	90 %	88 %	**81 %**
**InceptionResNetV2**	80 %	76 %	90 %	98 %	**86 %**
**InceptionV3**	88 %	86.00 %	94.00 %	98 %	**91.5 %**
**VGG-19**	24 %	76.00 %	76 %	72.00 %	**62 %**
**ResNet50**	82.00 %	78 %	92.00 %	96 %	**87 %**
**NASNetLarge**	84 %	78.00 %	90 %	94.00 %	**86.5 %**

**Table 3:** The Precision (Pre.) of each class for all pre-trained models.

Classifier	Pre.			
	N	P	S1	S2
**PPG-NET**	1.0	1.0	1.0	1.0
**Xception**	0.80	0.48	0.94	0.98
**MobileNetv2**	0.69	0.44	0.96	1.0
**DenseNet201**	0.46	0.32	0.71	1.0
**InceptionResNetV2**	0.63	0.45	0.82	0.96
**InceptionV3**	0.81	0.59	0.89	0.93
**VGG-19**	1.0	0.0	0.0	0.0
**ResNet50**	0.65	0.43	0.91	0.96
**NASNetLarge**	0.51	0.67	0.74	0.98

**Table 4:** The Sensitivity (Sen.) of each class for all pre-trained models.

Classifier	Sen.			
	N	P	S1	S2
**PPG-NET**	1.0	1.0	1.0	1.0
**Xception**	0.61	0.73	0.90	0.97
**MobileNetv2**	0.62	0.75	0.75	0.87
**DenseNet201**	0.58	0.38	0.81	0.67
**InceptionResNetV2**	0.63	0.47	0.82	0.92
**InceptionV3**	0.65	0.73	0.88	0.96
**VGG-19**	0.23	0.0	0.0	0.0
**ResNet50**	0.67	0.57	0.77	0.88
**NASNetLarge**	0.73	0.53	0.87	0.82

**Table 5:** The F1-score of each class for all pre-trained models.

Classifier	F1-score			
**PPG-NET**	N	P	S1	S2
	1.0	1.0	1.0	1.0
**Xception**	0.69	0.58	0.92	0.97
**MobileNetv2**	0.65	0.56	0.84	0.93
**DenseNet201**	0.51	0.34	0.76	0.80
**InceptionResNetV2**	0.63	0.46	0.82	0.94
**InceptionV3**	0.72	0.65	0.88	0.95
**VGG-19**	0.37	0.0	0.0	0.0
**ResNet50**	0.66	0.49	0.83	0.92
**NASNetLarge**	0.60	0.59	0.80	0.90

## 4 Discussion

The analysis of Table 2 shows that only PPG-NET achieved 100 % accuracy, indicating flawless classification performance on the test dataset. InceptionV3 and Xception are excellent performers, with 91.5 % and 90.5 % accuracies. MobileNetv2, ResNet50, NASNetLarge, and InceptionResNetV2 performed reasonably well at 87.5 %, 87 %, 86.5 %, and 86 % respectively. DenseNet201 had lower accuracies than the previous ones, which suggests some limitations in their ability to generalize or possibly issues with overfitting or underfitting depending on their training setup. VGG-19 notably underperformed with only 62 % accuracy, which is significantly lower than the others. This could indicate that the model's architecture is not suitable for the task, or it may have encountered issues such as failure to converge during training. True positives reveal how many instances each model correctly classified as belonging to its respective class. PPG-NET had the highest accuracy, also had the highest number of true positives, with each model correctly identifying 200 cases. Corresponding to their accuracies, models such as InceptionV3, Xception, and MobileNetv2 have decreasing TPs. NASNetLarge and DenseNet201, with their lower accuracies, also have fewer TPs at 173 and 162, respectively. VGG-19 with the lowest accuracy, had only 124 true positives, indicating a severe limitation in correctly classifying cases. Differences in the architecture's suitability for the dataset and task could be attributed to the variation in performance. For instance, architectures such as PPG-NET and Inception may capture features that are better suited for this classification problem than VGG-19. The distribution and balance of classes in the datasets may impact the results and behavior of each network. Each model's internal architecture (such as depth, complexity, or type of layers) may be more or less suitable for the spectral and time-based features in PPG signals. The level of tuning applied to each model can also have a significant impact on performance. Table 6 shows the parameters used to obtain the performance metrics of PPG-NET and all pre-trained networks.

**Table 6:** The tuning parameters for PPG-NET and all pre-trained models.

Fold Number	5
Epochs	100
Batch size	12
Optimizer	SGD
Learning Rate	0.01

For example, the learning rate, number of training epochs, and layer freezing/unfreezing during training are all critical factors. Therefore, they were fixed to make sure that the results are bench marked. Investigate why VGG-19 performed poorly, possibly by examining its training process, learning curves, and error analysis. Adjusting hyper parameters, particularly for models with moderate performance, could help optimize them. Future work may focus on exploring combining predictions from multiple models to improve the overall classification accuracy and robustness. A detailed examination may reveal that while some models excel, others lag behind, possibly due to differences in how they process and learn from the PPG data. Future work should focus on optimizing model parameters, exploring model ensembles, and possibly incorporating additional features or pre-processing steps to enhance classification performance. Table 2 offers a detailed breakdown of accuracy per class—Normal (N), Prehypertension (P), Stage 1 Hypertension (S1), and Stage 2 Hypertension (S2)—allowing for a comprehensive analysis of how each classifier handles different categories of blood pressure. This detailed examination helps pinpoint the specific strengths and weaknesses of each model. PPG-NET stands out with its uniform excellence, achieving 100 % accuracy across all classes and demonstrating robust ability to manage features specific to each class without bias. The PPG-NET provides models with a solid foundation and accelerates training by utilizing pre-trained weights from ImageNet and PPG datasets. PPG-NET's deep designs effectively extract hierarchical features. These topologies deal with vanishing gradients in deep networks by using depth wise separable convolutions to make processing go faster and keep connections alive. Combine these designs for robust feature extraction, enhancing complicated job performance. This performance improved accuracy by 8.5 %. The PPG-NET replaces fully connected layers, decreasing parameters while keeping the most important global properties. This eliminates overfitting and speeds up training and inference by making the model computationally efficient. The combined design creates a powerful model with outstanding accuracy, efficiency, and scalability for many applications. InceptionV3 and Xception also perform moderately well, although MobileNetv2 shows a slight drop in the P and S1 classes. ResNet50, does not show excellent performance as it may be expected due to the nature of the PPG scalograms. DenseNet201 and NASNetLarge exhibit below good performance, with NASNetLarge showing improved performance as the severity of hypertension increases, suggesting better handling of more advanced stages. On the lower end of the spectrum, VGG-19 struggles across all classes, with its highest accuracy being100 % in class N, indicating some capability in recognizing features of N stages but poor overall performance. The analysis reveals that models like PPG-NET and InceptionResNetV2 are highly effective across various hypertension stages and could be ideal for practical applications. MobileNetv2 and InceptionV3 could serve as good alternatives, potentially offering benefits in computational efficiency or easier implementation in mobile settings. The lower accuracy of some models in class P highlights the challenges in differentiating prehypertension from other stages. This suggests that further training or architectural adjustments may enhance their effectiveness. Additionally, the high variability in accuracy across classes could indicate overfitting in some models. This underscores the importance of using balanced and representative training data. Investigating why models like VGG-19 underperform could reveal valuable insights into their classification strategies and areas for improvement. This analysis of class-wise accuracy not only illuminates each model's capabilities but also aids in strategizing improvements and comprehending the behavioral dynamics of models across different stages of hypertension.

Upon analyzing the performance metrics of various pre-trained models across courses, we determined that each model achieved the highest F1 score, sensitivity (also known as recall), and precision. These are detailed in Table 3, Table 4, Table 5. The PPG-NET model stands out because it gets perfect scores in all metrics across all classes. This is because it uses depth wise separable convolutions, which are excellent at handling small to medium-sized datasets and extracting features from various types of data. Similarly, MobileNetV2 demonstrates nearly perfect scores but displays slightly lower precision and sensitivity in class P, likely due to its mobile device design, which balances efficiency and accuracy but may restrict detail capture. DenseNet201, despite being resilient against overfitting due to its feature reuse capability, scores the lowest in class P, possibly due to its intricate architecture, which may impede class-specific learning. Inception ResNetV2 also achieves perfect scores, benefiting from a hybrid architecture that combines Inception networks with ResNet, improving both error correction and feature extraction. InceptionV3, while excellent, exhibits slight decreases in precision and sensitivity in classes N and P, with its Inception modules intended to capture multi-scale features, although it may encounter difficulties with less distinct features. Despite its depth, VGG-19 performs inadequately, particularly in class P, where it fails. This may be due to overfitting or insufficient training on this specific dataset, resulting in significant class imbalances. ResNet50 generally performs well but shows some weaknesses in classes N and P, where its residual learning technique helps it avoid the vanishing gradient problem but may not capture all class-specific nuances. Lastly, NASNetLarge displays the weakest performance, especially in class P. The architecture is scalable and flexible, but it may not always generalize well across different data types or may require more extensive data to reach its full potential. In summary, models such as PPG-NET and InceptionResNetV2 emerge as top performers due to their sophisticated and balanced architectures. Conversely, models like VGG-19 and NASNetLarge exhibit notable weaknesses, possibly due to issues such as overfitting, under fitting, or misalignment with dataset specifics. Factors such as architectural design, the nature of the training data, and training procedures influence the performance of each model.

Table 7 is a comparative summary of the performance of various studies on blood pressure classification using PPG signals. The table includes details from different research papers that explore various methodologies, such as machine learning algorithms and signal processing techniques. It provides a snapshot of the diversity in methods and outcomes found in the current literature on PPG-based blood pressure classification. Each study uses different approaches to address the challenges of non-invasively monitoring blood pressure, providing a range of findings and technological innovations. It is worth noting that accuracy values may vary based on factors such as dataset size, signal quality, and the specific classification algorithms employed. After analyzing the methodology and results of the study on PPG signal-based classification of blood pressure stages, several key implications, limitations, and recommendations may be considered for future work. The successful application of deep learning models to classify blood pressure stages using PPG signals suggests a shift towards more accessible and non-invasive tools for monitoring hypertension. This advancement could significantly reduce reliance on traditional, more cumbersome blood pressure monitoring methods, enhancing patient comfort and compliance. Additionally, the use of simple, wearable technology equipped with PPG sensors allows for continuous monitoring of blood pressure levels. Continuous monitoring could play a crucial role in the early detection of hypertension, which is vital for preventing severe cardiovascular events. Moreover, data obtained from these technologies could be used to tailor healthcare plans to individual needs, promoting more personalized medicine. However, the study also reveals several limitations. For instance, the adaptation of some models, particularly DenseNet201 and VGG-19, is questionable due to their lower accuracy rates, which could lead to misclassifications and reliability issues in clinical settings. Several factors other than architecture may have influenced the results. First, oversampling balanced the dataset, but it may have added synthetic data points that the model can categorize, inflating performance measures. The model may have less challenge, especially in underrepresented classes, resulting in near-perfect accuracy. Second, the experimental arrangement strictly enforced subject-aware splitting; thus, there was no data leakage between the training and test sets, which could lead to overoptimistic results. Thus, the PPG-NET model would generalize without learning patient patterns in the training and test sets. Segmenting PPG signals into small intervals may also produce uniform data points, making it easier for the model to recognize recurring patterns within each class rather than blood pressure differences. The dataset may also not reflect real-world situations, including movement artifact noise, skin tones, and environmental surroundings. This constraint means the model's outstanding performance on this dataset may not apply to more difficult real-world circumstances. Therefore, the study's reliance on specific types of PPG data and the limited size of the dataset may affect the robustness and applicability of the findings across different demographics and physiological conditions. Furthermore, deploying deep learning models in real-world clinical environments presents complexities and requires significant resources, including training and adjustments to seamlessly integrate these systems into existing healthcare infrastructures. In conclusion, the results of this work show that PPG-NET, a compact convolutional neural network, can be used as a base for future wearable technologies equipped with PPG sensors to facilitate the continuous monitoring of blood pressure levels. Future work will increase the datasets and use multiple datasets from King Abdullah University Hospital which will be used to obtain the weights of the parameters. The work will also include the development of a novel light-sensitive PPG system for remote heart disease classification.

**Table 7:** Comparative accuracy performance of different studies on blood pressure classification using PPG signals.

Authors	Year	Method	Main Findings	Accuracy
Al Fahoum et al. [9]	2023	Applied CWT to extract PPG features and transfer learning to pre-trained deep learning models to classify BP into four classes	Achieved higher accuracy in BP classification using CWT and InceptionV3 pre-trained deep learning models transfer learning	99.5 %
Pankaj et al. [20]	2023	Modified the final three layers of a pre-trained deep neural network, GoogleNet, DenseNet, and AlexNet, to classify the TF spectrogram of the PPG (Fourier decomposition method (FDM)) signal into three classes	Achieved higher accuracy in BP classification using DenseNet	96.51 %
J Wu, H Liang, et al. [19]	2021	Deep Learning with Wavelet Transform	Achieved higher accuracy in BP classification using raw PPG signals and deep learning models.	90.5 %
Y Liang, Z Chen, et al. [38]	2018	Comparison of ECG and PPG	Found that PPG signals provide valuable data but are less effective alone in classifying different BP levels.	–
H Tjahjadi, and K Ramli [39]	2020	K-Nearest Neighbors (KNN)	Proposed a simple yet effective KNN method to classify BP levels without high-quality PPG signal requirements.	86.7 %
H Tjahjadi, K Ramli, and H Murfi, [40],	2020	Bidirectional LSTM	Utilized time-frequency analysis and LSTM to improve classification accuracy of BP using PPG signals.	92.3 %
S Haddad, A Boukhayma, et al. [41]	2021	Multi-Linear Regression	Focused on continuous monitoring and mapping of SBP and DBP using regression models from PPG data.	88.9 %

PPG-Net has demonstrated outstanding effectiveness on several benchmarks, often outperforming other cutting-edge models. The efficacy of the model stems from its ability to capture complex characteristics with a smaller number of parameters, leading to faster training and inference times. Adding residual connections to the intermediate layers also helps with the vanishing gradient problem, making it easier to train the network more thoroughly. The PPG-NET, which is being suggested, is a notable breakthrough in the domain of convolutional neural networks (CNNs) specifically designed for critical medical applications. It provides a superior and more streamlined alternative to traditional convolutional networks. PPG-NET is a useful tool for several computer vision problems because it can reduce the amount of computing required while still achieving excellent performance by utilizing depth wise separable convolutions. The novel design and strong performance of PPG-NET will greatly influence the advancement of future deep-learning architectures. For future speedy applications, the initial weights of the **PPG-NET** will be iteratively updated by training the network using both normal and abnormal PPG segments of 2.1 s obtained from patients at King Abdullah University Hospital using the experimental set up indicated in section 2.1. Data is being collected to fine-tune the network to ensure general and robust performance.

## 5 Conclusion and future perspectives

This study introduced and tested PPG-NET, a deep-learning model for blood pressure classification using PPG signals. This study examines deep-learning models for hypertension stage classification using wavelet analysis. PPG-NET's 100 % accuracy, precision, sensitivity, and F1-score across all categories demonstrated its aptitude for assessing PPG signals' complex properties. VGG-19 and NASNetLarge underperformed, with VGG-19 obtaining 62 % accuracy, indicating a task or dataset mismatch. These findings emphasize model selection in medical signal processing and show that deep learning can boost efficiency in specialized jobs.

Integrating scalograms, deep learning, and PPG signal processing offers the potential for non-invasive hypertension monitoring. Reliable cardiovascular health assessments provide quick and individualized care. The incorporation of biometric indicators and customization to diverse patient groups and conditions can significantly improve these models. PPG-based hypertension classification methods are transformative. The findings support that algorithms and technology will transform healthcare and drive precision-focused personal healthcare devices. Future research will improve underperforming models like VGG-19 and NASNetLarge to overcome this study's limitations. These models are essential since they are lightweight and hardware-compatible. Enhancements entail improving training procedures or creating adaptive architectures. A larger dataset with more data types and a higher sample size will improve model robustness and applicability across demographics and physiological situations. Future research will also examine whether PPG-NET's performance is due to more than its architecture. To assess the generalizability of the model, we will test it on additional diverse and challenging datasets to explore the potential overestimation of findings. The continued data collection will validate and upgrade the PPG-NET hardware iteratively.

These models must be evaluated in real life for practicality. Clinical pilots will identify operations or logistics issues with this technology. Engineers, data scientists, physicians, and healthcare professionals will work together to address deployment difficulties and guarantee that the technologies satisfy clinical goals and improve patient care.

## CRediT authorship contribution statement

**Amjed Al Fahoum:** Writing – review & editing, Writing – original draft, Validation, Supervision, Resources, Project administration, Methodology, Investigation, Funding acquisition, Formal analysis, Data curation, Conceptualization. **Ahmad Al Omari:** Visualization, Software, Formal analysis, Data curation. **Ghadeer Al Omari:** Visualization, Validation, Resources, Formal analysis, Data curation, Conceptualization. **Ala'a Zyout:** Software, Data curation, Conceptualization.

## Data availability statement

The datasets generated during and/or analyzed during the current study are available: https://www.nature.com/articles/sdata201820.

## Funding statement

This research was supported by the 10.13039/501100021772Deanship of Scientific Research and Higher Studies at 10.13039/501100006418Yarmouk University, grant number August 2024.

## Declaration of competing interest

The authors declare that they have no known competing financial interests or personal relationships that could have appeared to influence the work reported in this paper.

## Figures and Tables

**figs1:**